# Outcome of patients with idiopathic ventricular fibrillation and correlation with ECG markers of early repolarization

**DOI:** 10.1007/s00392-022-02125-9

**Published:** 2022-11-19

**Authors:** Benjamin Rath, Kevin Willy, Christian Ellermann, Patrick Leitz, Julia Köbe, Florian Reinke, Philipp Sebastian Lange, Gerrit Frommeyer, Lars Eckardt

**Affiliations:** Department of Cardiology II (Electrophysiology), University Hospital Münster, Albert-Schweitzer-Campus 1, 48149 Münster, Germany

**Keywords:** Early repolarization, Idiopathic ventricular fibrillation, S-ICD

## Abstract

**Background:**

Early repolarization pattern (ERP) has been associated with idiopathic ventricular fibrillation (IVF) and with cardiovascular mortality in the general population. As there is limited data about long- term outcome of IVF, the aim of our study was to observe ventricular arrhythmia (VA) recurrences in these patients and to identify a possible correlation of VA with ECG markers of early repolarization.

**Methods and results:**

We investigated 56 consecutive IVF patients who received an implantable cardioverter-defibrillator for secondary prevention. ERP was defined as a J-point elevation ≥ 0.1 mV in two or more contiguous inferior or lateral leads. Markers of early repolarization were present in 32.1% of cases with a preponderance of QRS slurring (77.8%). During a mean follow-up of 41.2 months, 11 patients (19.6%) received in total 18 adequate ICD-therapies. VF was most the common cause for ICDtherapy (61.1%) but monomorphic VT also occurred in four patients. Presence of ERP was associated with a significant trend towards arrhythmia recurrences. 38.9% patients with ERP received appropriate ICD-therapies whereas only 10.5% of patients without ERP had arrhythmia recurrence (*p* = 0.05). Inappropriate ICD-therapies occurred in seven patients (12.5%) with a non-significant trend towards a higher incidence in patients with a transvenous ICD (*p* = 0.15).

**Conclusion:**

A significant correlation between ERP and VA recurrences in patients with IVF could be observed. Though monomorphic VA also play a role in the studied IVF-population, our data support the use of the S-ICD in this collective.

**Graphical abstract:**

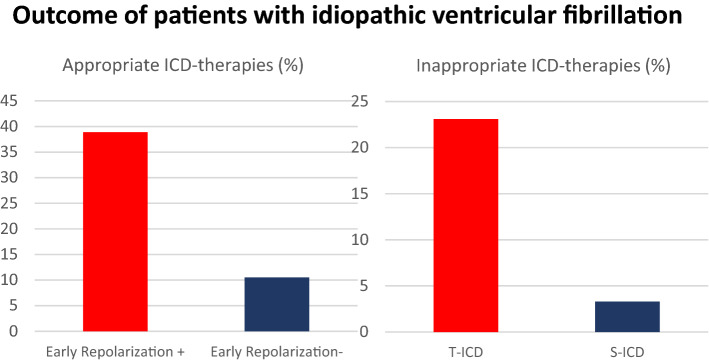

## Introduction

Sudden cardiac death (SCD) is a leading cause of death especially in developed countries and it is predominantly caused by ventricular arrhythmias (VA) in the context of coronary heart disease [1]. In contrast, idiopathic ventricular fibrillation (IVF) is characterized by spontaneous ventricular fibrillation in the absence of any structural heart disease, channelopathies, or transient causes of VA. IVF accounts for approximately 5–10% of all patients surviving an out-of-hospital cardiac arrest [2]. Several studies have identified an increased prevalence of early repolarization (ER) in patients with IVF [3, 4] but its prognostic impact for risk stratification as well as for long time outcome of these patients remains unclear. Except the implantation of an implantable cardioverter-defibrillator (ICD) and the empirical use of beta-blockers no established therapy for IVF exists [5].

Arrhythmia recurrence rates in IVF patients have been reported between 11 and 45% [6] and mortality rates have also been shown to be up to 5% over a 5-year period [7]. On the other hand, a relevant rate of inappropriate shocks in this especially young ICD-collective has been described [8]. This study from a large tertiary center sought to investigate the influence of early repolarization pattern (ERP) on arrhythmia recurrences in patients with IVF. Furthermore, we investigated outcome differences between patients equipped with a transvenous versus a subcutaneous ICD.

## Methods

This retrospective study involved an analysis of a consecutive cohort of 56 patients who underwent ICD implantation due to survived IVF at our center between 2008 and 2018. All patients received coronary angiography and cardiac magnetic resonance imaging (MRI) before ICD implantation without evidence of underlying structural heart disease. All MRI images were reevaluated before study inclusion to exclude patients with more recent diagnoses like mitral valve prolapse syndrome. No evidence for long-QT or short-QT syndrome was found in serial 12-channel-ECGs after cardiac arrest and in our outpatient clinic. Besides, Brugada syndrome and CPVT were ruled out by sodium channel blocker-test with ajmaline respectively exercise ECG. Patients with transient causes of VF such as electrolyte imbalance or drug intake were excluded as well.

In accordance with previous studies [3, 9], early repolarization pattern (ERP) was defined as a J-point elevation ≥ 1 mm (0.1 mV) in two or more contiguous inferior (II, III, aVF) or lateral leads (I, aVL, V4-6). ERP was divided into QRS slurring (a smooth transition from the QRS complex to the ST segment) or QRS notching (a positive J-deflection after the S wave). Patients were reassessed every 3 months, including device interrogation, in our outpatient clinic over a mean follow-up of 41 ± 35.45 months. All patients with at least one ECG with ERP, either during the initial in-hospital period or during follow-up in our outpatient clinic, were added to the ERP group. Clinical endpoints were defined as all-cause mortality as well as appropriate ICD therapies (VF or VT requiring antitachycardia pacing (ATP) or ICD-shock) and inadequate ICD therapies.

### Statistical analysis

SPSS software (version 26.0; SPSS Inc., Chicago, IL, USA) was used for statistical analysis and database management. For comparison of means, the Student’s *t* test was used for paired or unpaired observations, as appropriate. The *χ*^2^ test was applied for the comparison of proportions between groups. To determine independent predictors of arrhythmia recurrence, binary logistic regression models were used while adequate respectively inappropriate ICD therapies (yes; no) were set as the dependent variable. A *p*-value < 0.05 was considered statistically significant.

## Results

Our study population consisted of 56 patients who underwent ICD implantation secondary to idiopathic ventricular fibrillation. Baseline characteristics are listed in Table 1. The mean age was 37.76 years and 64.3% of patients were male. 30 patients (53.75%) received a subcutaneous ICD (S-ICD). Three of 26 patients (11.53%) who initially underwent transvenous ICD implantation (tv-ICD) were converted to an S-ICD due to lead complications during follow-up. Most patients (71.40%) were treated with ß-blockers. Two patients (3.6%) received amiodarone, one patient sotalol (1.8%), and three patients were treated with flecainide (5.4%).

**Table 1:** Baseline characteristics

	Total	Early Repolarization +	Early Repolarization -	*p*-value
*n*	56	18 (32.1%)	38 (67.9%)	
Male	36 (64.3%)	12 (66.7%)	24 (63.2%)	0.66
Age	37.8 ± 12.9	36.8 ± 13.3	38.2 ± 12.9	0.96
Follow-up (months)	41 ± 35.8	38.9 ± 37.2	42 ± 35.6	0.17
ICD-type				0.81
T-ICD	26 (46.2%)	7 (38.9%)	19 (50%)	
S-ICD	30 (53.8%)	11 (61.1%)	19 (50%)	
QRS-slurring	14 (25.0%)	14 (77.8%)	0	
QRS-notching	4 (7.1%)	4 (22.2%)	0	
Transient ERP	11 (19.6%)	11 (61.1%)	0	
Continuous ERP	7 (12.5%)	7 (38.9%)	0	
Medication	40 (71.4%)	15 (83.3%)	25 (65.8%)	0.14
Beta-Blocker	6 (10.7%)	3 (16.7%)	3 (7.9%)	0.13
AA Therapy	2 (3.6%)	1 (5.6%)	1 (2.6%)	
Amiodarone	3 (5.4%)	1 (5.6%)	2 (5.3%)	
Flecainide	1 (1.8%)	1 (5.6%)	0 (0.0%)	
Sotalol				

Early repolarization pattern on the surface ECG was present in 18 patients (32.1%) with a preponderance of QRS slurring (77.8%) compared to QRS notching (22.2%). Of these 18 patients, 11 (61.1%) had the continuous presence of ERP during follow-up. The mean follow-up was 38.9 months in patients with ERP respectively 42 months in patients without ERP. During follow-up, no deaths occurred in both groups. 11 patients (19.6%) received in total 18 adequate ICD therapies (16 ICD shocks, 2 deliveries of ATP). Ventricular fibrillation was the most common cause for ICD therapy (61.1%) but monomorphic or polymorphic VT also occurred in four (36%) respectively one patient. The presence of ERP was associated with a significant trend towards arrhythmia recurrences (Table 2). Seven of 18 patients with ERP (38.9%) received 12 ICD therapies over a mean follow-up of 38.9 months (20.68 therapies/100 patient-years), whereas only four of 38 patients (10.5%) without ERP had arrhythmia recurrence (4.5 therapies/100 patient-years) (*p* = 0.05). No difference between the specific patterns of ERP (QRS-slurring vs. QRS-notching), ECG localization (inferior vs. lateral) or variability of ERP (continuous vs. transient) with regard to arrhythmic burden was observed. The use of ß-blockers or antiarrhythmic drugs had no influence neither on arrhythmia recurrence (*p* = 0.14) nor on the type of VA (VF or VT) in this collective. In total, eight inappropriate ICD therapies occurred in seven patients (12.5%). The most common reason was inadequate detection of atrial arrhythmias or sinus tachycardia (62.5%) followed by T-wave-oversensing (25%) and oversensing due to lead failure (12.5%). ERP had no influence on inappropriate ICD therapies. There was a non-significant trend towards a higher incidence of inappropriate ICD therapies in patients with a tv-ICD (Table 3). Only one patient with an S-ICD received one inappropriate shock due to T-wave-oversensing, whereas six of 26 patients (23.08%) had in total seven inappropriate ICD therapies (*p* = 0.15). No device infections were reported, neither in S-ICD nor in the tv-ICD group.

**Table 2:** Results

	Total	Early repolarization +	Early repolarization −	
Pat. with appropriate ICD therapies	11 (19.6%)	7 (38.9%)	4 (10.5%)	0.05
Appropriate therapies in total	18	12	6	0.02
Appropriate therapies/100 patient years	9.4	20.7	4.5	
ICD shock	16	11	5	0.03
ATP delivery	2	1	1	0.62
MVT	5	3	2	
PVT	1	1	0	
VF	12	8	4

**Table 3:** Results 2 (transvenous ICD vs. subcutaneous ICD)

	Total	Transvenous ICD	Subcutanous ICD	
*n*	56	26 (46.4%)	30 (53.6%)	
Follow-up (months)	41 ± 35.8	58.2 ± 36.5	26.1 ± 27.9	0.01
Pat. with appropriate ICD therapies	11	7	4	0.59
Appropriate therapies in total	18	14	4	0.61
Pat. with inappropriate ICD therapies	7	6 (23.1%)	1 (3.3%)	0.15
Inappropriate ICD therapies in total	8	7	1	0.27
Inappropriate ICD therapies/100 patient-years	(3.7)	(4.8)	(1.5)	
SVT/sinus tachycardia	5 (62.5%)	5)	0	
T-Wave-Oversensing	2 (25%)	1	1 (100%)	
Oversensing due to lead failure	1 (12.5%)	1	0	

## Discussion

This analysis from a large tertiary center sought to investigate predictors of arrhythmia recurrence respectively inappropriate ICD therapies in patients with survived IVF. We observed a risk of appropriate ICD therapies of 19.6% over a mean follow-up of 41 months, which is comparable to other studies investigating IVF [2, 6, 7]. The risk of arrhythmia recurrence in the IVF collective seems to be in a similar range as other electrical heart diseases like Brugada syndrome [10] and thereby lower than in patients with structural heart disease. For example, in a secondary ICD prevention collective with coronary artery disease VA-recurrence rates of 69% over 3 years were described [11, 12]. No deaths occurred in our collective, which emphasizes the general favorable medium- and long-term outcome of IVF as long as the initial event is survived and an ICD is implanted. This is in line with the results of Chaundry et al. [5] who reported a total mortality of 4% in IVF survivors over 14 years follow-up with no cardiac cause of death.

We observed a significant correlation between ERP on the surface ECG and the recurrence of ventricular arrhythmias respectively appropriate ICD therapies. The prevalence of ERP in the general population varies between 1 and 5% [13, 14] with a higher degree in young individuals, especially males, athletes, and black people [15, 16]. ERP is associated with an increased risk of SCD [3, 17], especially in the context of structural heart disease [18]. Recently, an association between ERP in peripheral leads and higher risk of VA in Brugada syndrome has been reported as well [19]. In patients with survived SCD due to IVF, an increased prevalence of ERP between 20 and 42% has been described by several authors [3, 20–22] but with regard to VA-recurrences divergent data of the significance of ERP have been reported. Corresponding to our observation of an increased risk of appropriate ICD therapies in IVF patients with ERP, Haïssaguerre et al. [3] described a recurrence rate of VA of 41% in IVF patients with ERP versus 23% in patients without ERP. Honarbakhsh et al. [9] also reported a trend to a higher arrhythmia burden in patients with IVF and ERP and in particular a significant correlation between ERP with horizontal/depressed ST segments and VA-recurrence. In contrast, Chaundry et al. [5] did not find any influence of ERP on VA recurrence in a long time follow-up of 50 IVF patients with or without ERP. Likewise, Dalos et al. [20] also reported a similar rate of appropriate ICD therapies in IVF patients with and without ERP.

Of note, monomorphic ventricular tachycardia and not primary VF occurred in four out of 11 patients with VT/VF during follow-up although no arrhythmogenic substrate was found in cardiac magnetic resonance imaging. Similar findings were described in other IVF collectives, for example, by Dalos et al. [20]. A possible explantation for this observation in absence of a macroscopic substrate might be the presence of an electrophysiogical substrate. Haïssaguerre et al. [23] evaluated 24 IVF patients with multielectrode body surface recordings and endocardial and epicardial catheter mapping. The authors found localized structural alterations especially in the right ventricle as well as a high incidence of Purkinje triggers as a possible substrate for reentry. One could imagine that these alterations might also promote VT especially under the influence of beta-blockers or antiarrhythmic drugs. However, we did not find a correlation between type of VA recurrence and medication in this cohort.

More than half of the patients in our study received a subcutaneous device representing, to our knowledge, one of the largest described IVF collective with an S-ICD. Over the whole cohort, inappropriate ICD therapies occurred in 12.5% of patients, which correlates with the results of other IVF registries [9, 24]. We observed a trend towards a higher rate of inappropriate ICD therapies in patients with transvenous ICD compared to subcutaneous devices. No deaths, ineffective therapies or need for conversion to a transvenous ICD were observed in the S-ICD-collective. However, a relevant part of the inappropriate ICD therapies in the tv-ICD group occurred in the first years of the observed period. Probably a significant proportion of these ICD- interventions, especially for sinus tachycardia, might be prohibited by a more conservative “state of the art” ICD-programming [25]. Nevertheless, our data support the use of the S-ICD in IVF according to its feasibility in primary electrical heart disorders which has been described before [24, 26].

### Study limitations

Due to the rarity of IVF, we conducted a retrospective analysis. Our median follow-up of 41 months might be too short to estimate a reliable long time prognosis, especially if different ICD systems (S-ICD vs. tv-ICD) were implanted. Most ECGs were acquired early after the initial resuscitation event. Hence, drugs or intensive care therapy might influence a part of the observed ECG abnormalities.

## Conclusion

Early repolarization pattern occurs frequently in patients with idiopathic ventricular fibrillation. Overall, the recurrence rate of VT/VF seems to be low compared to other secondary prevention ICD populations. However, a significant correlation between ERP and ventricular arrhythmia burden could be observed in this cohort. Interestingly, fast monomorphic VA might also play a role in the studied IVF population, but nevertheless our data support the use of the subcutaneous ICD in this collective.
